# Computational Advances in Ionic Liquid Applications for Green Chemistry: A Critical Review of Lignin Processing and Machine Learning Approaches

**DOI:** 10.3390/molecules29215073

**Published:** 2024-10-26

**Authors:** Brian R. Taylor, Nikhil Kumar, Dhirendra Kumar Mishra, Blake A. Simmons, Hemant Choudhary, Kenneth L. Sale

**Affiliations:** 1 Joint BioEnergy Institute, Emeryville, CA 94608, USA; 2 Department of Biosecurity and Bioassurance, Sandia National Laboratories, Livermore, CA 94551, USA; 3 Department of Bioresource and Environmental Security, Sandia National Laboratories, Livermore, CA 94550, USA; 4 Biological Systems and Engineering Division, Lawrence Berkeley National Laboratory, Berkeley, CA 94720, USA

**Keywords:** Density Functional Theory (DFT), biomass processing, lignocellulosic biorefineries, lignin depolymerization, reactive force fields (ReaxFF), solvent screening, quantum chemistry, multiscale modeling

## Abstract

The valorization and dissolution of lignin using ionic liquids (ILs) is critical for developing sustainable biorefineries and a circular bioeconomy. This review aims to critically assess the current state of computational and machine learning methods for understanding and optimizing IL-based lignin dissolution and valorization processes reported since 2022. The paper examines various computational approaches, from quantum chemistry to machine learning, highlighting their strengths, limitations, and recent advances in predicting and optimizing lignin-IL interactions. Key themes include the challenges in accurately modeling lignin’s complex structure, the development of efficient screening methodologies for ionic liquids to enhance lignin dissolution and valorization processes, and the integration of machine learning with quantum calculations. These computational advances will drive progress in IL-based lignin valorization by providing deeper molecular-level insights and facilitating the rapid screening of novel IL-lignin systems.

## 1. Introduction

The need for sustainable alternatives to fossil-based resources has driven significant interest in lignocellulosic biomass as a renewable feedstock for biofuels and chemicals. Lignin, which constitutes 15–35% [1] of this feedstock, offers significant potential as a source for high-value products such as biofuels [2], materials [3], and pharmaceuticals [4]. Unlocking this potential is critical to the economic viability of the biobased economy [5].

The valorization of lignin presents several unique challenges. It resists solubilization and depolymerization and is chemically complex [2]. Furthermore, the structure and composition of lignin vary significantly depending on its biomass source and extraction method, resulting in different types of technical lignins (e.g., Kraft, lignosulfonate, Organosolv, Soda) with distinct properties. These different types of technical lignins often have different associated purities, molecular weights, and functional groups [6,7,8,9]. This variability complicates efforts to develop universally applicable conversion technologies. Most current biorefineries often combust the lignin rather than turn it into high-value products [10,11,12,13].

Ionic liquids (ILs) are promising solvents for lignin dissolution and processing, offering a potential solution to the challenges associated with lignin depolymerization. ILs are effective biomass solvents because of their thermostability, ability to dissolve a wide range of substances, tunable nature, and low vapor pressure [14,15,16]. Their tunability is due to the large number of possible cation–anion combinations. However, it is costly, labor-intensive, and time-consuming to conduct experimental screenings of ILs due to the wide range of cation and anion combinations. Computational screening, on the other hand, has proven to be a viable option for multi-scale screening of “notable” solvents in biomass processing [17,18,19,20]

The vast improvement in computational resources provides an opportunity for computational studies to play a large role in identifying amenable ILs. However, significant challenges remain in applying computational methods to IL-based lignin valorization. These include accurately representing lignin’s structural heterogeneity, balancing computational cost with predictive accuracy, and translating molecular-scale insights to process-level optimization. Additionally, the scarcity of standardized, high-quality experimental data for training and validating computational models poses a barrier to developing robust predictive tools.

This review aims to critically assess the current state of computational methods for understanding and optimizing the IL-based lignin dissolution and valorization processes reported since 2022. We examine the strengths and limitations of various approaches, from quantum chemistry to machine learning. The scope of this review excludes experimental methods, industrial-scale applications, and non-IL-based solvent systems. By highlighting recent advances and identifying key knowledge gaps, we seek to provide a roadmap for future research that will support the design of IL-based systems that allow for lignin dissolution and valorization, ultimately contributing to the development of sustainable biorefinery processes and a circular bioeconomy.

## 2. Screening of Ionic Liquids (ILs) Based on Modeling

Conducting experimental tests on all the potential cation–anion combinations in ILs is difficult due to their multitude. To effectively handle this level of complexity in ILs, computational screening methods are required. Because lignin is heterogeneous and has different bonding patterns, it is difficult to say how it will solvate. When simplified lignin models are used in simulations, they might not accurately represent how real lignin made from biomass behaves, which lowers the accuracy of the screening data. Despite advances in IL screening for lignin solvation, there are no general rules to predict which ILs are most effective in deconstructing biomass. The absence of such guidelines leads to trial-and-error approaches and slows down the discovery of optimal solvents. Accurately predicting the critical thermodynamic properties (such as activity coefficients, excess enthalpy, and solubility parameters) for IL-lignin systems is challenging, as these depend on specific interactions that vary widely among ILs and model lignin structures. Robust theoretical techniques are required for efficient screening of the extensive range of ILs for lignin dissolution. Recent improvements have facilitated the implementation of several predictive methodologies for simulating the thermodynamic parameters of IL-containing systems. Earlier prominent techniques encompass the Perturbed Chain Statistical Associating Fluid Theory (PC-SAFT) [21], which was used to calculate the thermodynamic properties of different homologous series of ILs based on the bis(trifluormethylsulfonyl)imide anion ([NTf_2_]^−^); group contribution approaches [22] were explored for estimating critical properties, normal boiling temperatures, and acentric factors for IL; Quantitative Structure–Property Relationships (QSPRs) [23] were used to estimate the infinite dilution activity coefficients for organic solutes in IL; Monte Carlo (MC) molecular simulations [24] explored the understanding the gas solubility in IL; Molecular Dynamics (MD) simulations [25] were performed to understand the pure and mixed gas absorption in the ionic liquids; and the Conductor-like Screening Model for Real Solvents (COSMO-RS) [26,27] model was applied for screening of solvents for multiple applications such as asphaltene dispersion, gas absorption, and extraction of biologically active compounds [28,29,30]. It follows that the COSMO-RS model is one of the most effective predictive approaches for screening ILs for various applications due to its ability to quickly process vast IL datasets. Experimental investigations might be conducted with much less time, money, and effort if ILs could be screened using predictive techniques that only needed a small number of input parameters.

**Figure 1:**
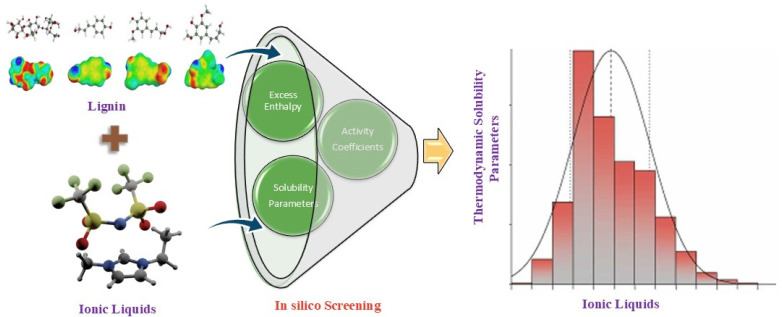
The schematic diagram for screening of solvents for lignin dissolution.

While emerging methods like COSMO-RS and machine learning (ML) have shown promise, scaling these models to screen thousands of ILs with diverse lignin models requires significant computational resources and optimization to ensure practical, cost-effective screening [31,32]. Translating predictions into real-world applications is often hindered by the complexity of IL synthesis, cost, and the need for reliable experimental data to find the best solvents. COSMO-RS has previously been successfully used to predict thermodynamic properties such as solubility in IL systems, activity coefficients (γ), and excess enthalpy (HE). Several research articles emphasize the ability of these parameters to screen ILs for lignin solvation [17,33,34]. The basic schematic diagram for in silico screening of ILs for lignin solvation is shown in Figure 1. Various researchers used basic lignin model compounds, such as p-coumaryl alcohol, sinapyl alcohol, and coniferyl alcohol, to represent the lignin structure. The main links between the structural units of lignin are β-O-4 (β-aryl ether), β-β (resinol), and β-5 (phenylcoumaran) [35]. As a result, researchers developed various lignin models, which consist of three basic units (H, G, and S) with some basic linkages, to investigate the impact of lignin structure on dissolving behavior. However, the current understanding of ILs and lignin models remains relatively basic, posing a challenge in formulating comprehensive guidelines for identifying the best solvents for lignin.

Mohan et al. conducted an extensive screening of 5670 ILs, generated from 63 cations and 90 anions, to assess their effectiveness in lignin dissolution [17]. The lignin structure was built by joining all the major lignin linkages (β-O-4, β-β, 4-O-5, α-O-4, and β-5) present in the native lignin, and the screening results were validated using the experimental data. They identified that anions like acetate, methyl carbonate, glycinate, alaninate, and lysinate were particularly efficient in breaking down lignin, especially when paired with cations such as tetraalkylammonium ([TAA]^+^), tetraalkylphosphonium ([TAP]^+^), and pyridinium. Specifically, ILs containing [TAA]^+^ and [TAP]^+^ showed great promise as solvents due to their low viscosity, favorable Hansen solubility parameter alignment, and enhanced hydrogen-bonding basicity. Yu et al. further studied 3886 ILs using COSMO-RS. They used the 19 model structures of lignin (monomers, dimers, and trimers) for predictions and validated them through experimental solubility tests [30]. The monomeric, dimeric, and trimer structures of lignin fail to correctly represent the lignin molecule due to the absence of several different linkages found in lignin. Additionally, Kamlet–Taft parameters were used to clarify the mechanisms involved in lignin dissolution, highlighting the importance of IL screening in optimizing lignin processing. In a further extension of this work, Mohan and coworkers explored the molecular mechanisms behind the effectiveness of cholinium lysinate ([Ch][Lys]) IL in lignin processing [36]. Using MD simulations alongside COSMO-RS, they determined that [Ch][Lys] serves as a promising solvent for lignin dissolution. This preference is attributed to multiple factors, including increased molecular interactions, extensive hydrogen bonding, a higher dissociation constant, and reduced viscosity. These characteristics collectively enhance the efficiency of [Ch][Lys] in the dissolution of lignin.

### 2.1. Limitations of Solvent Screening for Lignin

#### 2.1.1. Complexity of Lignin Structure

Most computational screening models simplify lignin’s structure to make simulations feasible, but this can lead to inaccurate predictions. It is challenging to make a plausible model of how lignin interacts with ILs because it is a very heterogeneous polymer with many different functional groups and bonding patterns. Simplifying lignin into small model compounds (such as guaiacol or syringol) does not capture the full complexity of the polymer [31]. Lignin is a complex and diverse biopolymer that poses substantial obstacles for characterization, depolymerization, and applications. Elaborating on the discourse regarding the “complex parameters” of lignin necessitates examining the following elements that enhance its intricacy:

##### Structural Heterogeneity

The structural elucidation of lignin has revealed that 50% of lignin’s constituents contain aromatic rings. Lignin consists of three principal monolignol units: p-coumaryl alcohol, coniferyl alcohol, and sinapyl alcohol. These units constitute a stochastic network of ether (C-O) and carbon–carbon (C-C) bonds. The irregular polymerization results in significant structural diversity, complicating the prediction of lignin’s reactivity in processes or its solubility in solvents. The composition of these monomers differs throughout plant species and even within various tissues of the same plant, contributing to its complexity [37].

##### Branching and Cross-Linking

Lignin, in contrast to linear polymers, exhibits extensive branching characterized by several inter-unit connections, including β-O-4 (alkyl aryl ether), β-5 (phenylcoumaran), 5-5 (biphenyl), and β-β (pinoresinol), among others. Likewise, the most abundant linkage in both softwood and hardwood lignin is the β-O-4 linkage. These linkages provide a three-dimensional matrix that inhibits depolymerization, leaving lignin more recalcitrant than other biopolymers such as cellulose and hemicellulose.

##### Bond Variability

Lignin comprises robust C-C bonds and more reactive C-O bonds, necessitating targeted cleavage strategies. The existence of many functional groups (hydroxyl, methoxy, carbonyl, etc.) hinders selective bond cleavage. The reaction of various bonds is contingent upon variables such as the solvent, catalyst, and temperature, complicating the regulation of the breakdown of lignin and resulting in erratic product distributions.

##### Polydispersity

Lignin has a strong polydisperse molecular weight distribution, indicating a broad spectrum of molecular weights and structural changes within any specific sample. The polydispersity influences both the physical qualities and chemical reactivity, as varying molecular weights react differently to processing conditions [38].

##### Functional Group Diversity

Lignin possesses an abundance of functional groups (hydroxyl, methoxy, phenolic, and carbonyl), with their distribution exhibiting variability. This variation affects lignin’s reactivity, solubility, and interactions with other substances (e.g., catalysts, solvents). Furthermore, phenolic hydroxyl groups are crucial for antioxidant action, yet they can complicate depolymerization due to their resistance to certain chemical transformations.

##### Lignin–Cell Wall Interactions

Lignin is not found in isolation; it is closely associated with other cell wall constituents, especially cellulose and hemicellulose. These interactions create a rigid framework that hinders the extraction and separation of lignin. The matrix inherently resists enzymatic and chemical destruction, necessitating extreme conditions or innovative solvents for effective fractionation of lignocellulosic biomass [39].

##### Source Variability

The composition and structure of lignin vary considerably based on the plant source (e.g., hardwood, softwood, grasses). Softwood lignin is predominantly composed of guaiacyl (G) units, whereas hardwood lignin comprises both guaiacyl (G) and syringyl (S) units. These variations modify lignin’s reactivity and require distinct methodologies for its conversion or valorization.

Future research should focus on improving computational power or algorithms to allow simulations of larger, more complex lignin structures without compromising accuracy.

#### 2.1.2. Solubility vs. Selectivity

While some ILs are excellent at dissolving lignin, their selectivity toward different lignin fractions and derivatives remains a challenge. Current screening methods often prioritize solubility over selectivity, potentially resulting in inefficient lignin fractionation or subpar product quality. Future research should focus on ILs that are both highly soluble and selective for specific lignin components [40].

#### 2.1.3. Computational Screening and Predictive Models

While computational tools such as COSMO-RS and MD simulations can predict IL-lignin interactions, their accuracy remains limited. There is a need for improved algorithms and more comprehensive datasets that account for the variability in lignin structure and IL chemistry. Future efforts should aim at integrating machine learning models with quantum calculation data from first-principle calculation to enhance screening efficiency. Fixing these issues will significantly advance future research in creating ILs for lignin valorization. This will result in biorefinery processes that work better and last longer. In the context of IL screening for lignin, computational screening and predictive models are becoming vital tools. However, to improve their accuracy and efficiency, we must address several challenges. One of the primary challenges in developing accurate predictive models for IL-lignin interactions is the scarcity of high-quality “standardized” experimental data. Most computational models, including machine learning (ML) approaches, require extensive datasets to predict IL performance. Because there are not any standardized experimental data for how lignin dissolves or depolymerizes in different ILs, it is challenging to make reliable models. Future work should focus on generating large, consistent datasets that capture both the structural variability of lignin and the wide range of IL chemistry.

#### 2.1.4. Accuracy of Force Fields and Interaction Parameters

MD simulations and other computer methods utilize pre-established force fields to illustrate the interactions between lignin and ILs. The complexity of IL chemistry and lignin variability often limits the accuracy of these force fields, which is critical. Current force fields might not capture subtle interactions such as hydrogen bonding or π-π stacking accurately. We need better machine-learned force fields that are made for ILs interacting with biopolymers like lignin in order to make MD simulations more accurate.

#### 2.1.5. Computational Cost

High-level computational methods, like ab initio MD (AIMD) or quantum mechanical (QM) calculations, can give us a lot of information about how IL and lignin interact, but they are time- and computer-intensive. Large-scale screening, which involves evaluating hundreds or thousands of ILs, makes these methods impractical. To enhance the overall efficiency of the screening process, it could be beneficial to strike a balance between the costs associated with each step’s calculations and how accurate they are. We could achieve this by developing hybrid methods that blend faster or less detailed models with occasional high-precision calculations.

#### 2.1.6. Uncertainty in Thermodynamic Predictions

We often use tools like COSMO-RS to guess the thermodynamic features of IL-lignin systems, like their activity coefficients, how well they dissolve, and their Gibbs free energy. However, these methods often have limitations for complex mixtures or systems with strong intermolecular interactions and averaging predictions might not accurately capture the specific behavior of the system [41]. Furthermore, predicting the impact of solvent impurities can be challenging. Temperature variations and the impact of IL recycling on lignin solubility continue to pose challenges. These uncertainties can lead to suboptimal IL selection during screening. Future efforts should focus on refining these thermodynamic models and incorporating uncertainty quantification to improve their predictive capabilities.

In light of the aforementioned limitations, a conclusion table elaborating on the constraints of the solvent screening for lignin is presented in Table 1.

## 3. Ionic Liquids for Lignin Valorization

The process of breaking down lignin through the use of ILs is a developing area of study aimed at transforming this intricate biopolymer, which is plentiful in plant biomass, into useful low-molecular-weight substances [42]. The application of ILs in lignin depolymerization has become increasingly popular because of their distinctive characteristics, such as adjustable acidity and basicity, excellent thermal stability, and re-usability to some extent, which makes them perfect for promoting chemical reactions [43].

A lot of research using Density Functional Theory (DFT) and MD simulations has provided crucial insights into the reaction mechanisms, energy barriers, and dynamics of interactions for complex reactions [44,45,46,47,48]. Recent investigations have explored the use of ILs as solvents and catalysts for the depolymerization of lignin and model compounds, utilizing DFT and MD simulations to reveal the mechanisms by which these substances dissolve and decompose lignin into valuable compounds. This theoretical framework allows for the optimization of conditions to enhance the yield and selectivity of targeted phenolic monomers throughout the depolymerization process. Disputes in the discipline frequently center on the effectiveness and ecological consequences of employing ILs in contrast to conventional techniques like reductive and oxidative depolymerization. Although some researchers advocate for the selectivity and reaction control benefits of ILs, others express apprehensions about their overall sustainability for their use in large-scale applications [49].

Furthermore, as discussed in the previous section, the complex nature of lignin’s composition and structure requires continuous research to thoroughly understand how various ILs affect the depolymerization results and to enhance computational models for precise predictions. With the ongoing evolution of computational techniques, the combination of DFT and MD simulations is anticipated to significantly improve the comprehension and optimization of lignin depolymerization, leading to more sustainable methods for biomass valorization [50].

The comprehension of lignin depolymerization in the presence of ILs has consistently been a captivating area of research; however, insights into the process via QM methods have been limited when compared with experimental investigations. Nevertheless, there have been limited theoretical investigations aimed at enhancing comprehension of the process. This builds on several earlier investigations aimed at elucidating the cleavage mechanism of traditional C-O bonds in lignin when exposed to different combinations of ILs [45,46,51]. The earlier research focused on elucidating the catalytic mechanism for the depolymerization of lignin model compounds in the presence of ILs. This was achieved by examining the thermodynamics of the process, investigating the activation energy barriers, and analyzing the bonding parameters within the lignin-IL complex system. Building on a similar methodology, a recent investigation conducted by Liu et al. examined the electrocatalytic depolymerization of lignin into valuable chemicals, presenting a promising avenue for sustainable biorefineries [52]. The study utilizes dispersion-corrected DFT calculations to elucidate the electrocatalytic reduction mechanism associated with the depolymerization of lignin model dimers and oxidized lignin. The computational study was completely focused on understanding the depolymerization of β-O-4-based model compounds. The calculations clarify the role of the trimetallic ILs-PdNiBi catalytic system in promoting the generation of a crucial radical intermediate and the release of products. The anticipated energies of the reaction pathways validated the function of ILs with metals in achieving total substrate conversion and elevated yields of phenols and acetophenones by reducing the activation energy barriers, thereby aiding in C-O bond cleavage. The development of C-O bond cleavage can enhance lignin valorization and utilization by facilitating the selective breakdown of lignin into valuable chemicals while preserving the functional groups necessary for material applications. For example, demethylation, a specific form of C-O bond cleavage, involves breaking the bond between the methyl group and oxygen in methoxy (-OCH_3_) substituents on aromatic rings. The process results in increased phenolic content and the reactivity of lignin fragments serving as valuable precursors for materials, phenolic resins, polyurethanes, etc. [53].

With recent advancements in the conversion of lignin into materials, DFT calculations have been critical in elucidating the electron transport process and material’s electronic structure. Chen et al. recently published a study in which they used lignosulfonate as a carbon source from biomass to make ultrahigh-energy density supercapacitor electrodes. Calculations were performed to gain a deeper insight into the process of electron transport. Simulations grounded in DFT also contributed to a deeper understanding of the electronic and ionic characteristics, as well as the adsorption energies of the fabricated supercapacitor electrodes. This kind of research concerning material synthesis involving lignin and ILs is experimentally accessible, yet it remains limited from a theoretical standpoint, owing to the challenges posed by lignin’s complicated structure that requires resolution [54]. DFT-based optimization of lignin modifications and studying structure–property relationships can be achieved and would be of great help in the future. By revealing lignin’s complex molecular behavior, these multidisciplinary approaches help speed the development of lignin-based materials and sustainable technologies.

The integration of QM calculations with ML techniques has proven to be a significant advantage in this domain, accelerating the prediction and validation of reaction mechanisms while minimizing computational effort and associated expenses. A recent investigation by Ding et al. effectively utilized machine learning alongside QM calculations to predict the bond cleavage efficiency of a set of approximately 10^3^ possible ILs on the β-O-4 bond of guaiacyl glycerol-β-guaiacyl ether (GGE), achieving a strong correlation between the predicted bond dissociation energy and the experimental yield of guaiacol, emphasizing the reliability of the ML model [55]. The study pinpointed imidazolium-based cations paired with tyrosine anions as ideal candidates for future applications in the precise cleavage of lignin. This approach showcases an innovative method that bypasses the necessity for intricate activation barrier energy calculations while greatly minimizing the time required for evaluating potential ILs. DFT calculations have been helpful to generate a lot of useful molecular descriptors that can be used by the ML models. In another study performed, DFT-generated datasets were used as the input parameters for training ML models to understand the photocatalytic cleavage of C-C bonds in lignin-derived structures [56]. The extent of datasets for training ML models is restricted by the high computational cost of DFT. The generalization of ML models to a variety of systems may be impeded by the scarcity of data. Furthermore, while ML can expedite predictions, it frequently compromises accuracy in comparison to DFT. Nevertheless, these studies were limited to model monomeric/dimeric molecules and there is an urgent need to expand these calculations to various model polymeric lignin structures to gain further insights. The primary challenge in combining these methods is the delicate balance between the reliability of results and computational efficiency, especially when dealing with a combination of ILs and lignin systems.

On the contrary, methods such as MD simulations have emerged as a key tool in deciphering the intricate process of lignin depolymerization. These simulations offer a deeper understanding of the structural dynamics and interactions with solvents. In a study by Hackenstrass et al., the authors conducted classical MD simulations to uncover intricate details about the structural dynamics of lignin dimers, illustrating how different linkages influence lignin conformation and its interaction with water [44]. The structures used for predictions were based on dimers composed of common linkages found in lignin, including 4-O-5, 5-5, α-O-4, β-1, β-5, β-β, and β-O-4. This information is essential for predicting the solubility and reactivity of lignin in polar solvents. The study reveals that the β-O-4 linkage exhibits unique characteristics regarding its conformational flexibility and water interaction, which are essential for its solubility and depolymerization processes. Similar predictions can be noted in the presence of ionic solvents. In a recent study, the behavior of lignin with multiple chains was investigated within the 1-ethyl-3-methylimidazolium acetate and water system. The analysis of simulation trajectories revealed that lignin formed an aggregated complex in pure water, preserving its hydrophobic regions. Lignin chains were observed to disentangle and extend their conformation at varying concentrations of ILs in the system. The authors investigated the interaction patterns of multiple lignin chains with guaiacyl decamer in the presence of ILs, which can offer insights into the dissolution phenomenon of lignin at the atomic level [18]. Additionally, reactive force field (ReaxFF) simulations have emerged as an essential asset to the community. The ability to realistically replicate the dynamics, reactivity, and effects of solvents on the solute is a further advantage of using ReaxFF-based simulations [57]. As a result of the ease of simulating small chemical structures, ReaxFF-based simulations have been conducted on lignin-derived structures. Since it is difficult to replicate the complex and heterogeneous nature of lignin, model compounds are frequently used by researchers. A model compound of lignin serves as a simplified representation of a particular structural unit, allowing for a focused investigation of the specific bonds or functional groups present in natural lignin. Nonetheless, a few studies have investigated the use of this method for the thermo-oxidation of lignin or lignin-based products. ReaxFF simulations were employed in a study to investigate thermal decomposition in an oxygen environment for models that represented the most prevalent linkages found in softwood. This revealed the reaction pathways of dominant reaction products [58]. A ReaxFF-based simulations were used to investigate the oxidative stabilization of softwood lignin for carbon fiber production. It focuses on lignin fragments derived from coniferyl alcohol units, revealing that the 5-5 linkage exhibits the highest reactivity towards cyclization and dehydrogenation, which is crucial for forming the rigid connections necessary for stabilization [59]. The structures used in this study consist of five to six coniferyl alcohol units connected through different linkages (namely 4-O-5, 5-5, α-O-4, β-1, β-5, β-β, and β-O-4). Two structures were preferred, including 9 lignin fragments containing 5 monolignols, 9 lignin fragments containing 6 monolignols, and 396 oxygen molecules. The cost-effectiveness associated with ReaxFF in comparison to conventional MD simulations facilitated the utilization of large structures. In a recent study, Ahmad et al. presented a technique for identifying products and their pathways in thermo-oxidation reactions through ReaxFF simulations, aiming to elucidate the dynamics and reactivity of the thermo-oxidation of two model structures of modified lignin [60]. Nonetheless, employing ReaxFF simulations to comprehend the utilization and valorization of lignin with ILs requires significant advancement, particularly in the development of accurate force fields for extensive lignin structures and ILs.

Recently, a new theoretical strategy named “Advanced real-time molecular sensing strategy” was introduced, which is claimed to be capable of capturing the dynamic nature of lignin reactions, including delignification and de/repolymerization. The work integrated the combination of DFT, AIMD, and kinetic Monte Carlo (kMC) simulations, constituting a comprehensive framework for assessing various lignin properties. This approach was capable of providing real-time molecular level information about dissolved lignin chains during delignification, including molecular weight distributions and the S/G ratio under diverse reaction conditions. However, this study was not conducted with ILs, but it can surely be a usable synergistic integration of theoretical approaches with experimental data for the understanding of the lignin systems in presence of ILs and their potential for sustainable biorefineries in the future [61]. A comprehensive grasp of this subject can aid in the formulation of a cost-effective process for lignin dissolution using ILs and even depolymerization, ultimately facilitating an improved biorefinery operation.

### 3.1. Challenges

Despite the advances made using DFT and MD simulations, several challenges remain. One significant challenge is the computational cost associated with simulating large lignin structures. While DFT is excellent for small systems, its computational expense becomes prohibitive for larger systems, necessitating the use of simplified lignin models, which may not fully capture the complexity of real lignin.

One significant challenge lies in the accuracy of the models used to represent lignin’s complex structure. Different approaches, such as using repeating building blocks or a stochastic approach to vary linkages and monomers, aim to create representative lignin models. However, these models often come with inherent uncertainties, especially regarding the linkage distributions, which can have a margin of error of up to 5% [62]. Such discrepancies can lead to variations in the predicted behavior of lignin during depolymerization processes. Furthermore, the MD simulations are influenced by the time step chosen, with common practices employing smaller time steps. However, lower Metropolis temperatures or increased Monte Carlo (MC) events might improve simulated statistics but also increase computational complexity.

Additionally, accurately modeling ILs is challenging due to the wide range of interactions, such as hydrogen bonding, van der Waals forces, and electrostatic interactions, present in these systems. High-level quantum chemical methods are often required to model these interactions accurately, making simulations computationally intensive. Moreover, the dynamic behavior of ILs, especially when mimicking the real experimental conditions, adds another layer of complexity to the simulations. Reactive simulation techniques can be useful to understand the dynamic structures of lignin and ILs, as well as being capable of tracking the reaction in the presence of solvents and catalysts. Polarizable force fields were introduced in conjunction with reactive force field simulations to address the simulation concerns related to ILs, and they may be more accurate in capturing local electronic effects than non-polarizable force fields. Their limited DFT use to date is certainly due to increasing computing cost or difficulty in implementation, despite their capacity to mimic more delicate electronic characteristics [63,64]. The major advantage of the method of ReaxFF is its ability to show how chemical reactions occur with no preassigned connectivity, which is useful for a number of dynamic studies in the chemistry and materials science fields. However, the method has limitations, especially when applied on larger structures. These are the advanced processes of parameterization, lower precision in relation to QM calculations, and difficulties in interpreting the simulation results of complex systems. Also, it should be noted that while ReaxFF allows carrying out simulations that are many times larger than QM calculations allow, there are also limitations in practical terms for the size of the model and for the duration that can be simulated [57]. Table 2 presents a comprehensive overview of the simulation parameters discussed in this section of the paper, which covers the types of lignin structures, ILs, and simulation methodologies that are pertinent to the valorization of lignin.

### 3.2. Future Perspectives

Numerous prospective avenues may aid in addressing these challenges and enhancing the theoretical comprehension of lignin depolymerization through the use of ILs.

#### 3.2.1. Integration of ML

By training models on existing data from DFT and MD simulations, researchers could forecast the properties of new ILs and the structural properties of lignin without relying on resource-intensive quantum chemical calculations. This method has the potential to greatly accelerate the identification of novel ILs for the depolymerization of lignin.

#### 3.2.2. Advanced Multi-Scale Modeling

The combination of DFT and MD within a unified multi-scale framework would enable researchers to effectively capture both the electronic and dynamic behaviors involved in the depolymerization of lignin in ILs. This method may offer a comprehensive perspective on the depolymerization process and contribute to the formation of more effective ILs. The development of accurate reactive force fields for lignin and ILs can also be a solution to this field, as they can capture the reactive dynamics of the system at a lower computational cost in comparison to classical MD simulations. Another method to be worked on intensively is DFT-based transition state calculations for complex molecules such as lignin, which are time-intensive, challenging, and susceptible to convergence issues. In order to resolve this matter, the implementation of multi-level hybrid methods that integrate semi-empirical approaches with DFT has the potential to expedite these calculations, thereby enhancing accuracy and efficiency.

#### 3.2.3. Selective Catalysis

Formulating tailored ILs that focus on particular lignin linkages (e.g., C-O, and C-C) while reducing unwanted side reactions will be essential for enhancing lignin depolymerization. The computational data analyzed through DFT and MD simulations are quite limited and have primarily been applied to a specific group of ILs, which restricts the overall understanding and should be expanded.

## 4. ML Methods for Lignin Processes

ML techniques are increasingly being applied to optimize and predict the outcomes in lignin valorization processes using ILs, as also highlighted in the previous sections. This emerging approach offers significant potential to accelerate research and development by reducing the need for time-consuming and costly experimental work across various scales—from molecular interactions to process development.

The synthetic flexibility of ILs lends itself to large datasets. As a result, they provide ample opportunity for ML methods. As a result, a growing amount of research has been published on the use of ML for ILs [65]. The use of ML for ILs in general has been comprehensively reviewed before [66]. Furthermore, the use of ILs with ML for applications such as CO_2_ capture [67] and thermo-chemical properties [68] has also been reviewed. But in this section of the review, we focus specifically on ML applications for lignin extraction and valorization.

At the molecular level, ML is proving valuable for predicting the fundamental properties crucial for IL-based lignin processing. More sophisticated approaches using graph-based genetic algorithms and graph neural networks have been developed for solvent design in lignin-first biorefineries. This framework designed numerous solvents with high potential for lignin solubilization, demonstrating solubilities between 20 and 60 wt% across different lignin types, including Kraft lignin from softwood, organosolv lignin from hardwood, and herbaceous lignin from corn cob by mild acidolysis [19]. Complementing this, other researchers have developed predictive toolsets that combine Hansen solubility parameters, COSMO-RS predictions, and ML models to identify effective lignocellulosic pretreatment solvents. These approaches not only predict solvent effectiveness but also provide insights into the dissolution mechanism through quantum chemical calculations [32]. Furthermore, recent work has utilized explainable ML models based on Kamlet–Taft and polarity parameters to predict and optimize lignocellulose pretreatment efficiency, offering both high predictive accuracy and the interpretability of solvent properties crucial for lignin processing [69].

Moving to catalyst design, ML is also being applied to optimize both chemical and biological catalysts for lignin processing. While not specifically focused on ILs, work on using ML to engineer cytochrome P450 enzymes for the optimal bioconversion of lignin fragments demonstrates the potential of this approach [70]. Similar techniques could be adapted to design catalytic ILs or to optimize IL-enzyme systems for lignin valorization. In the realm of chemical catalysis, ML models have been developed to predict the catalytic activity of ILs for cleaving the β-O-4 bond in lignin. The best model achieved an R2 value over 0.93, allowing rapid screening of potential IL catalysts [55]. This work highlights how ML can accelerate the discovery and optimization of IL-based catalytic systems for lignin depolymerization.

Considering larger scales, ML is being applied to optimize reaction conditions and predict process outcomes. Random forest regression has been used to predict bio-oil yield and char yield from IL-catalyzed lignin depolymerization, achieving R2 scores of 0.91 and 0.94, respectively [71]. These types of predictive models can help researchers identify promising reaction conditions without extensive experimental trials. The power of ML in uncovering non-obvious relationships in lignin processing has been further demonstrated. Using algorithms like XGBoost to model lignin hydrogenolysis revealed that parameters such as the lignin-to-solvent ratio and catalyst pore size had large impacts on outcomes [72]. This type of insight can guide more targeted experimental design and process optimization. At the process development level, Bayesian optimization has been applied to simultaneously optimize multiple lignin properties in an IL-based biorefinery process. This allowed efficient navigation of the multi-dimensional process parameter space and identification of optimal conditions, significantly accelerating the development and scale-up of IL-based lignin valorization processes [73].

An important consideration in applying ML to lignin valorization is the selection and engineering of appropriate descriptors or input features. While many studies use readily available parameters like IL cation/anion identities and reaction conditions, more sophisticated approaches are emerging. Various molecular representation techniques for ILs have been reviewed, highlighting the potential of natural language processing methods that can learn directly from SMILES strings or other text-based molecular notations [66]. Building on this, Transformer Convolutional Neural Networks operating on SMILES inputs have been found to outperform traditional descriptor-based models for predicting IL melting points [74]. This suggests that advanced text-based ML models could offer improved predictive power for IL properties relevant to lignin processing. The use of explainable ML models is particularly noteworthy. Models have been developed to predict lignocellulose pretreatment efficiency based on Kamlet–Taft and polarity parameters [69]. Such approaches not only achieve high predictive accuracy but also provide insights into the relative importance of different solvent properties. This type of interpretable ML model is crucial for gaining scientific insights and building trust in ML predictions for lignin valorization processes.

Beyond reaction optimization, ML is also being applied to analytical techniques relevant to lignin research. ML models have been used to predict the lignin content in poplar wood using Raman spectroscopy data [75]. This showcases how ML can enhance rapid characterization methods, potentially enabling real-time monitoring and control of lignin processing for downstream processing.

Despite these advances, challenges remain in applying ML to IL-based lignin valorization. The available datasets are often small and fragmented, and the complex, heterogeneous nature of lignin poses difficulties for developing universally applicable models. Proper validation is crucial, as the random splitting of limited IL datasets can lead to overly optimistic performance estimates [74].

Looking forward, integrating ML with high-throughput experimentation could enable rapid closed-loop optimization of IL-lignin systems. Incorporating mechanistic knowledge into ML architectures may improve accuracy and interpretability. There is also significant potential in applying ML to analyze spectroscopic and analytical data from IL-lignin reactions, potentially yielding new chemical insights. ML methods have already demonstrated clear value in accelerating research on IL-based lignin valorization across multiple scales, from molecular design to process optimization (Table 3). As datasets grow and algorithms improve, ML is likely to play an increasingly central role in this field.

## 5. Conclusions

Given the need to valorize lignin for a sustainable biorefinery, this review highlights the expanding influence of computational methods and machine learning approaches in lignin processing using ILs. The substantial progress in computational resources, including the integration of ML techniques, has significantly reduced the timeline of optimizing deconstruction through data-driven methods to gaining atomic- and molecular-level insights into complex lignin-IL interactions. This review additionally identifies the major research gaps and potential future directions to tackle unknown challenges in lignin valorization using ILs. For instance, the need for the (a) extensive experimental validation of simulated/predicted data, (b) introduction of complexity in lignin structure, (c) standardization of simulated data and open access data-sharing, and (d) integration of ML with high-throughput experimentation. Ongoing advancement and expansion of computational methods to enhance lignin dissolution and valorization, particularly through next-generation predictive paradigms, are crucial for establishing the fundamental science needed to develop and optimize effective lignin conversion processes. In summary, realizing the full potential of lignin as a renewable feedstock will require continued collaboration between data scientists, chemists, and chemical engineers to develop ML tools tailored for the unique challenges of IL-lignin systems.

## Figures and Tables

**Table 1:** Conclusion table elaborating on the constraints of the solvent screening for lignin.

Factor	Limitation	Impact on Lignin Screening
Lignin Structural Variability	High structural diversity of lignin not fully represented	Inconsistent solvent performance across different lignin structural types
Lack of Universal Solvent	No single solvent effectively dissolves all lignin types due to their structural diversity	Screening results vary depending on the source of lignin
Process Optimization Complexity	Optimizing conditions like temperature and pressure is resource-intensive and may not yield consistent results	Makes difficult for the benchmarking of results with available data
Computational Expense	High computational cost for large-scale solvent screening with real lignin	Constrains the number of solvents and conditions evaluated
Cosolvents System	Neglect co-solvent system or presence of water	Incomplete or limited understanding of lignin dissolution in real-world scenarios
Predictive Models	Over reliance on specific models without experimental validation	Potential inaccuracies in predicting lignin solubility

**Table 2:** Details about the lignin structure, ILs, and simulation methods discussed related to the valorization of lignin using ILs or solvents.

Types of Lignin or Lignin-Derived Structures	Ionic Liquids	Simulation Methods	Reference
Phenyl p-hydroxycinnamate	[Bmim][FeCl_4_]	DFT calculations	[45]
Guaiacyl glycerol-β-guaiacyl ether	[C_3_SO_3_Hmim][HSO_4_]	DFT calculations MD Simulations	[51]
Phenethoxybenzene 2-Phenoxyacetophenone	[Pyr_13_][NTf_2_] [Pyr_14_][OTf] [N_1113_][NTf_2_]	Periodic DFT calculations	[52]
Guaiacyl glycerol-β-guaiacyl ether	Combination of 29 cations and 33 anions, around 1000 possible ILs	DFT calculations; ML Technique	[55]

**Table 3:** ML applications in IL-based lignin valorization.

Key Findings/Outcomes	ML Methods	Reference
Predicted solvent properties	XGBoost, RF, GPR, SVR, KNR	[69]
Predicted IL catalytic activity	ANN, SVR, LR, RF, GBDT	[55]
Predicted bio-oil yield and char yield from IL-catalyzed depolymerization	RF	[71]
Optimized hydrogenolysis outcomes using lignin-to-solvent ratio and catalyst pore size	LightGBM, XGBoost, and CatBoost	[72]
Optimized multiple lignin properties in IL-based biorefinery process	Bayesian optimization	[73]
Predicted IL melting points using SMILES inputs	Transformer Convolutional Neural Networks	[74]
Predicted lignin content in poplar wood	SVR, RF, LightGBM, CatBoost, XGBoost	[75]

Abbreviations: Extreme gradient boosting (XGBoost), Random forest (RF), Gaussian process regression (GPR), Support vector regression (SVR), Linear regression (LR), K-neighbor regression (KNR), Artificial neural network (ANN), Gradient boosting decision tree (GBDT).
